# Missing single nucleotide polymorphisms in Genetic Risk Scores: A simulation study

**DOI:** 10.1371/journal.pone.0200630

**Published:** 2018-07-19

**Authors:** Miguel Chagnon, Jennifer O’Loughlin, James C. Engert, Igor Karp, Marie-Pierre Sylvestre

**Affiliations:** 1 Centre de recherche du Centre Hospitalier de l’Université de Montréal, Montréal, Québec, Canada; 2 Department of Social and Preventive Medicine, School of Public Health, University of Montréal, Montréal, Québec, Canada; 3 Departments of Medicine and Human Genetics, McGill University, Montréal, Québec, Canada; 4 Department of Epidemiology and Biostatistics, Schulich School of Medicine and Dentistry, Western University, London, Ontario, Canada; South Texas Veterans Health Care System, UNITED STATES

## Abstract

Using a genetic risk score (GRS) to predict a phenotype in a target sample can be complicated by missing data on the single nucleotide polymorphisms (SNPs) that comprise the GRS. This is usually addressed by imputation, omission of the SNPs or by replacing the missing SNPs with proxy SNPs. To assess the impact of the omission and proxy approaches on effect size estimation and predictive ability of weighted and unweighted GRS with small numbers of SNPs, we simulated a dichotomous phenotype conditional on real genotype data. We considered scenarios in which the proportion of missing SNPs ranged from 20–70%. We assessed the impact of omitting or replacing missing SNPs on the association between the GRS and phenotype, the corresponding statistical power and the area under the receiver operating curve. Omission resulted in a larger bias towards the null value of the effect size, a smaller predictive ability and greater loss of statistical power than proxy approaches. The predictive ability of a weighted GRS that includes SNPs with large weights depends of the availability of these large-weight SNPs.

## Introduction

The potential to understand the genetic underpinnings of complex diseases has been extended considerably with the advent of the human genome project [1]. However, the effect of one single-nucleotide polymorphism (SNP) on a complex phenotype is typically small, explaining only a small proportion of the variability in the phenotype. Since Purcell and al. reported that combining “risk alleles” of selected SNPs into an “aggregate risk score” predicted schizophrenia and bipolar disorder [2], there has been growing interest in what is now referred to as Genetic Risk Scores (GRS) [3–6] for a variety of different phenotypes.

GRS are usually created in a discovery sample and analysed in a target sample. First, SNPs are selected based on their nominal *P*-value for a specific phenotype observed in a genome-wide association study (GWAS) as a discovery sample. The GRS is then computed as the weighted sum of the risk alleles of selected SNPs, where weights are defined by the marginal effect size of each SNP. Second, the association between this GRS and the phenotype is studied in another sample, called the target sample. When weights are similar, a weighted GRS with all weights set to one is equivalent to an unweighted GRS with a simple count of risk alleles [7].

Two approaches are commonly used to choose SNPs in a GRS. The first selects only SNPs significant at a GWAS level (*P*-value < 5x10^-8^) in the literature or in a discovery dataset. The resulting GRS is based on a relatively small number of SNPs (often < 100). This approach usually provides information on the precise genetic and biological mechanisms underpinning the phenotype but at the expense of lower predictive ability [8]. The second approach aims to maximize the predictive ability of the GRS, and selects hundreds of SNPs using a liberal *P*-value threshold such as 0.001 [4, 9, 10]. This approach may require both discovery and target samples in hand if the discovery GWAS did not include reports of all SNPs under a 0.001 threshold.

SNPs selected in the discovery sample may not all be available in the target dataset (e.g., when the discovery sample is drawn from the literature, when the datasets were genotyped with different technologies, or when the SNP call rate was too low in the target dataset [11]). Approaches to deal with this issue include omitting the SNP in the GRS computation [12], replacing it with a proxy SNP that is available in the dataset [13] or imputing unavailable SNPs [14] using stand alone software (e.g., IMPUTE [15], minimac [16], BEAGLE [17]) or software available through a public server (https://imputationserver.sph.umich.edu) [18]. For each approach, imputation quality can be measured using the expected value of the squared Pearson correlation coefficient (r^2^) between the true and estimated allele counts. The r^2^ coefficient is estimated by the ratio of the observed variance of the allele count after imputation and the expected variance based on a binomial distribution under Hardy-Weinberg equilibrium (HWE) [18].

Proxy SNPs are usually selected among nearby SNPs determined to be in linkage disequilibrium (LD) with the unavailable SNP, using a reference panel of similar ethnicity [13, 19]. As in imputation, the quality of a proxy SNP can be measured by the r^2^ between the unavailable SNP and the proxy SNP from the reference data panel. Under the assumption of no population sub-stratification and HWE, the proxy r^2^ corresponds to the expected value of r^2^ between the true and the estimated allele counts in the target sample. Proxy SNPs can easily be found with web-based applications such as SNAP [20] or LDlink [21].

Depending on the sample size, genetic imputation can be an efficient method for replacing a large number of missing SNP values [4]. However, for a GRS based on a limited number of SNPs, or when only a few SNPs are missing, several authors have used the proxy approach because of its simplicity and rapidity [6, 13, 19, 22, 23]. Regardless of method, however, there will be SNPs for which imputation is either not feasible or of poor quality, or SNPs for which no proxy is available, leading to omission of the unavailable SNPs in the GRS. Goldstein et al. investigated the impact of omitting imputed SNPs on the predictive ability of a large GRS for a binary phenotype according to different imputation quality thresholds [4]. The predictive ability of the GRS was not affected by omission of SNPs with poorer quality imputation. However, a small decrease in effect size was observed in an extreme case in which SNPs were restricted to those with an imputation quality of r^2^ > 0.9 (i.e., 2332 of 7387 SNPs were omitted) [4].

While omission and proxy approaches are routinely used in smaller GRS, their impact on GRS performance has not been systematically investigated. In this article, we use statistical simulations to compare the impact of omission versus proxy approaches on the estimated association between weighted and unweighted GRS based on a limited number of SNPs and a binary phenotype. We also investigated the impact on the predictive ability of the GRS. We used a published GRS for coronary heart disease (CHD) [6] as a template to simulate plausible scenarios for a weighted GRS in which data on more than one SNP are missing and in which the quality of the proxy SNPs varies.

## Methods

A statistical simulation study using real genotype data was conducted to assess and compare the impact of omitting unavailable SNPs or using proxy SNPs, on GRS performance. The simulation study with 10 000 iterations involved four steps:

### Simulation

#### Data generation

We used genetic data from the 404 participants in the Northern European panel of the 1000 Genomes project (without the CEU sub-population) to represent genetic data with a common LD structure [24]. The CEU sub-population was set aside as an independent reference panel for the proxy approach. For simplicity, we restricted the pool of SNPs to the region 1 to 6-Mb aligned to the ‘+’ strand from chromosomes 1 to 10 with a minor allele frequency (MAF) ≥ 5% in HWE. An offset of 1-Mb prevents an edge effect in the proxy SNP search. To avoid using SNPs for which no proxies were available, we further restricted the pool to SNPs for which data on: (i) an excellent proxy (*r*^2^ ≥ 0.9); (ii) a very good proxy (0.8 ≤ r^2^ < 0.9); and (iii) a good proxy (0.6 ≤ *r*^2^ < 0.8) were available in the CEU sub-population. The number of SNPs retained for simulation ranged from 2236 to 7140 for chromosomes 1 to 10.

A binary phenotype was simulated from 50 SNPs (50 corresponds to the number of SNPs in the GRS in the CHD example) [6]. We randomly selected five SNPs that were not in LD per chromosome, assuming a causal genetic model. Let *Y*_*j*_ represent the phenotype status of participant j (*Y*_*j*_ = 0 or 1). Let *X*_*ij*_ = 0, 1, 2 represent the number of risk alleles of participant j for SNP i. The vector of causal SNPs for participant j is *X*_*j*_ = (*X*_1*j*_,*X*_1*j*_,…,*X*_10*j*_)^*T*^. The disease risk (over a risk period) corresponding to a weighted GRS for participant j is given by:

$$logit[P(Y_{j}|X_{j})]=\mu +\sum_{i=1}^{50}\beta_{i}X_{ij}$$ (1)

where the weight *β*_*i*_ is the log odds ratio (OR) for a one-risk-allele increase in SNP *i*. We considered both weighted and unweighted GRS. Assuming the effect sizes of the SNPs are similar, an unweighted GRS can be used in which *β*_*i*_ = *β*, leading to

$$logit[P(Y_{j}|X_{j})]=\mu +\beta \times \sum_{i=1}^{50}X_{ij}$$ (2)

Unweighted GRS is thus a count of the number of risk alleles ($\sum_{i=1}^{50}X_{ij}$) and *β* corresponds to the log OR for an increase of one risk allele.

In simulations corresponding to a causal unweighted GRS model, we considered scenarios in which common weights were set at values corresponding to the log OR, ranging from 1.05 to 1.1. Simulations corresponding to a causal weighted GRS used 50 weights corresponding to those reported for the GRS for CHD [6]. These weights corresponded to a median OR of 1.07 with an interquartile range of 0.4. Weights included three extreme weights for which the ORs (1.51, 1.45 and 1.29, respectively) were more than 1.5 interquartile range above the third quartile of the OR distribution. These extreme weights allowed us to study the impact of unavailable SNPs when contributions to the GRS were much larger than other SNPs.

The constant μ in Eqs (1) and (2) defines the disease risk in the absence of risk alleles, which corresponds to *exp*(*μ*)/[1 + *exp*(*μ*)]. For a specific set of weights, the μ parameter was set using preliminary simulations to ensure a marginal relative frequency of Y = 1 of approximately 50%. The predicted risk based on this genetic risk model *p*_*j*_ was calculated for each participant with the inverse logit function. The simulated phenotype for participant *j* was obtained by random generation of a Bernoulli distribution with probability *p*_*j*_.

#### Missing data generation

To reflect plausible scenarios with missing data on SNPs [6, 25, 26], we randomly set 20%, 30%, 50% or 70% of SNPs as missing.

#### GRS construction

Four alternate GRS were computed in each scenario: (i) *GRS omission* refers to the GRS computed with available SNPs only; (ii) *GRS with excellent proxy SNPs* refers to the GRS computed by replacing missing SNPs with proxy SNPs of excellent quality; (iii) *GRS with very good proxy SNPs;* and (iv) *GRS good proxy SNPs* refers to GRS computed in the same way but with proxy SNPs of very good or good quality, respectively. For each SNP with a missing status, three proxy SNPs of differing quality (i.e., excellent, very good, good) were obtained with SNAP from the 1000 Genomes Pilot 1 SNP dataset of the CEU sub-population with a distance limit of 500 kb [20].

#### GRS performance evaluation

**Fig 1:**
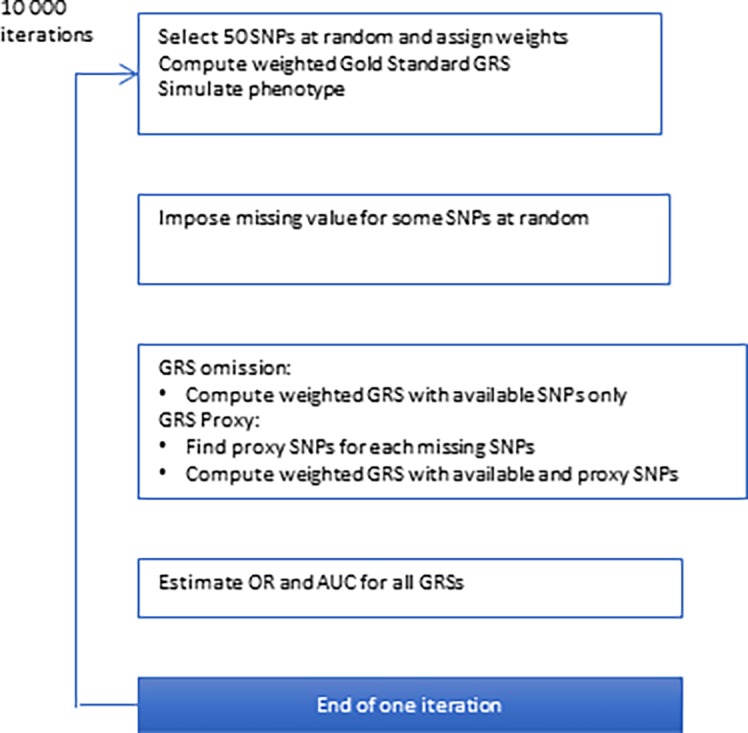
Schematic overview of the algorithm used for simulation of weighted GRS.

For all GRS, the OR for a one-standard-deviation increase in the GRS was estimated as a measure of the GRS and phenotype association. The area under the receiver operating curve (AUC) was estimated as a measure of the predictive ability of the GRS for the phenotype (Fig 1).

### Statistical analysis

The prevalence of the disease and GRS values were described in each scenario using means, standard deviations, and minimum and maximum values. The impact of omitting or replacing unavailable SNPs was assessed in each scenario by comparing the performance of the corresponding gold standard GRS (using all original SNPs) with that of the four alternate GRS. We first considered the distribution of Pearson correlations between the gold standard GRS and the four alternate GRS from each of the 10 000 simulated datasets. Because of the left-skewed distribution of correlations, the results were described using box plots. For each scenario, we reported the median, 5^th^, 25^th^_,_ 75^th^, and 95^th^ percentiles of the OR estimating the association between the GRS and the phenotype. ORs were used to facilitate interpretation, but the estimated regression coefficients can be obtained using a log transformation. To study the statistical power to detect a given OR at α = 0.05, we computed the percentage of p-value that were <5% over all simulations. Similarly, we reported the median AUC to investigate the predictive ability of the GRS. Simulations were performed using R version 3.3.1 and packages GenABEL version 1.8–0, genetics version 1.3.8.1 and pROC version 1.9.1.

## Results

Over all simulations, the mean (SD) prevalence of disease risk was 49.3% (4.4%) (minimum 26.7%, maximum 68.1%). The mean of the gold standard unweighted GRS was 26.95 (1.72) (minimum 19.40, maximum 34.79). The mean of the gold standard weighted GRS was 2.44 (0.20) (minimum 1.65, maximum 3.24).

### Impact on the GRS value

**Fig 2:**
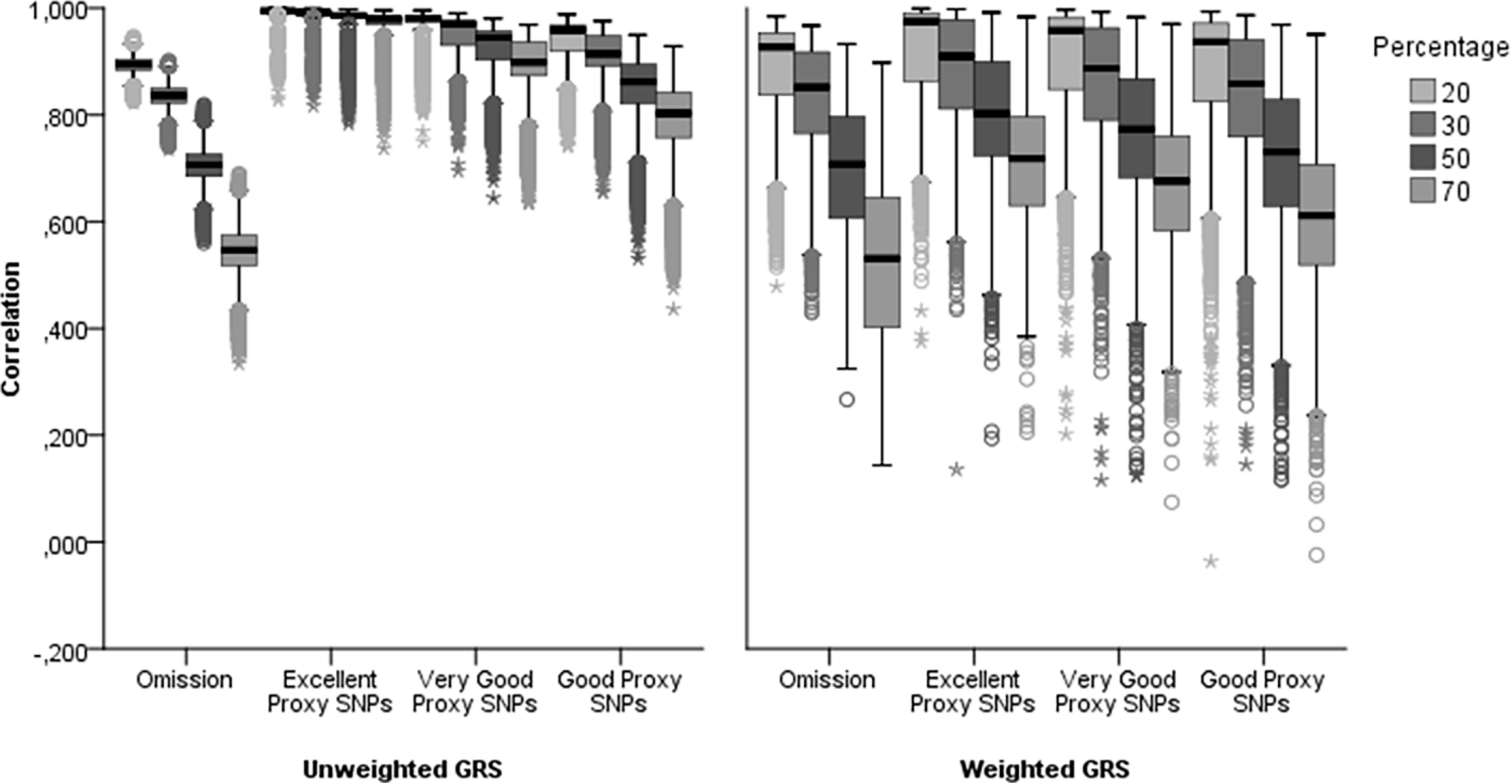
Impact of omission of the unavailable SNPs or replacement by proxy SNPs on the correlation between the GRS and gold standard GRS. Results for unweighted GRS and weighted GRS correspond to different scenarios. The interpretation must be made separately and not contrast.

The correlations between the gold standard GRS and each alternate GRS over the 10 000 simulations decreased when: (i) the proportion of unavailable SNPs increased, and (ii) when the quality of proxy SNPs decreased (Fig 2). Independent of the proportion of unavailable SNPs, the median correlation reported for GRS obtained after omission of unavailable SNPs was similar for weighted and unweighted GRS. However, the wider interquartile range for weighted GRS suggest greater variability than for unweighted GRS. When 20% of SNPs were unavailable, correlations with the gold standard GRS were all >0.8 for unweighted GRS; 84% of the correlations for weighted GRS were >0.8 (S1 Table). At any given proportion of unavailable SNPs, the median correlation was lower with omission than with the proxy approach, regardless of whether weights were used. Similarly, at any given proportion of missing SNPs and holding the quality of proxy SNPs constant, the median correlation was higher in unweighted compared to weighted GRS and the interquartile range was always wider for weighted GRS.

The relationship between the mean MAF of unavailable SNPs and the correlations between the gold standard unweighted GRS and the estimated GRS are shown in S1 Fig. There was a slight negative association between the mean MAF and the correlations. S2 Fig suggests that the mean MAF of unavailable SNPs and the correlations between the gold standard weighted GRS and the estimated GRS are not associated. However, stratifying the results by the availability of SNPs with the largest weights indicates a positive strong relationship between the availability of the SNPs with the largest weights and the correlation between the gold standard weighted GRS and the estimated GRS (S2 Table). Regardless of the proportion of unavailable SNPs and the approach used, the median correlation was ≥ 0.82 when the three SNPs with the highest weights were available, and ≤ 0.73 when they were unavailable.

### Impact on effect size estimation

**Fig 3:**
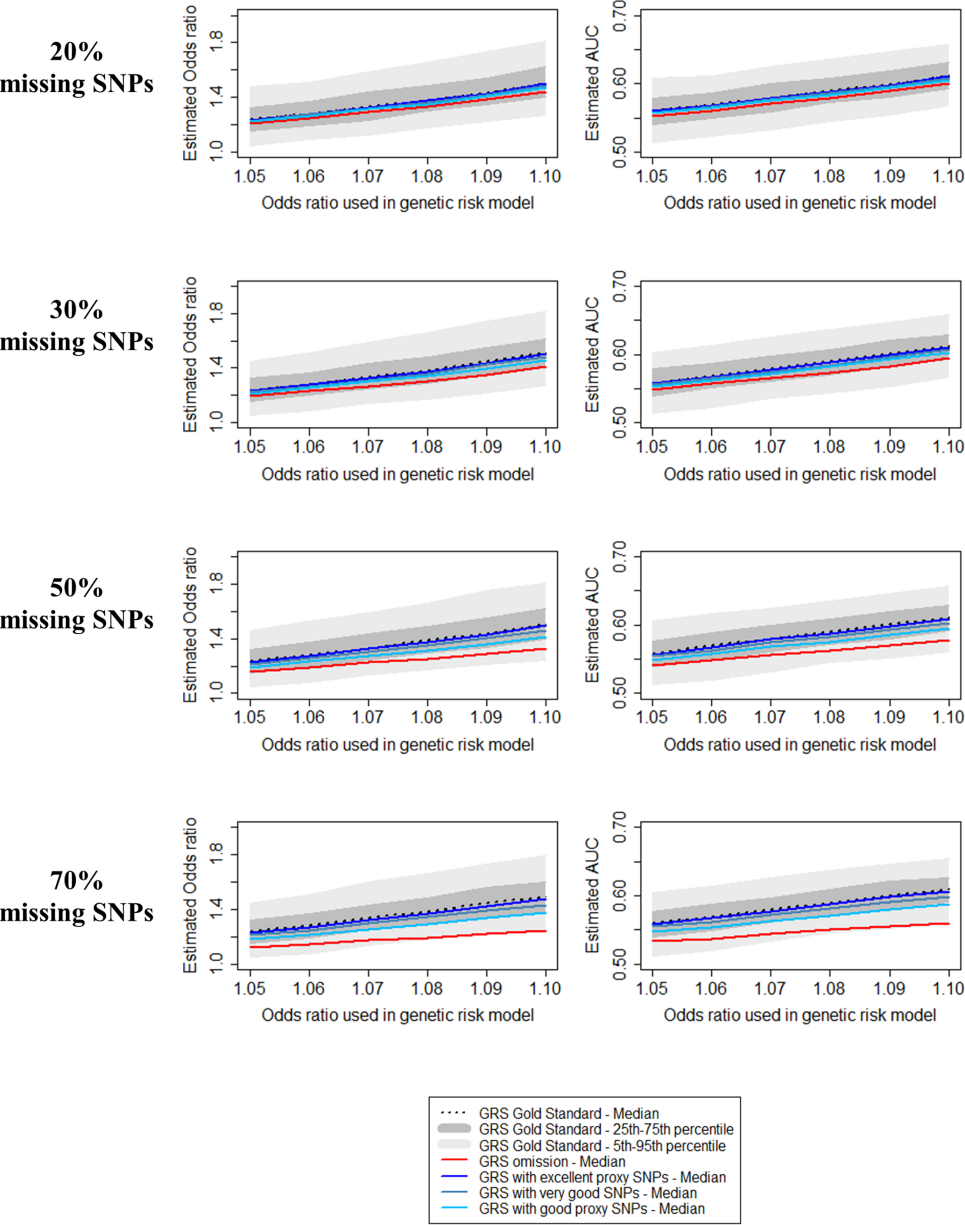
Impact of omission of the unavailable SNPs or replacement by proxy SNPs on effect size estimation and predictive ability of the unweighted GRS for the phenotype.

The left-hand side panels of Fig 3 show the estimated ORs as a function of the effect size OR = exp(*β*) in Eq 1 for the genetic causal model for the unweighted gold standard GRS, for the omission and proxy approaches when 20%, 30%, 50% and 70% of SNPs are missing. Regardless of scenario or approach used, the estimated ORs were below the median OR of the gold standard GRS. Specially, when the proportion of missing SNPs was 20%, the median OR for each approach was between the 25^th^ and 75^th^ percentile of the OR of the gold standard GRS. Underestimation increased with the proportion of unavailable SNPs and size of the OR = exp(*β*) used to simulate the data, but remained very small when excellent proxy SNPs were used. The median OR estimated using the proxy approach remained in the interquartile range of the OR from the gold standard GRS when the proportion of unavailable SNPs was <70% and the OR = exp(*β*) used to simulate the data was at its highest value (1.10).

With omission, the median estimated OR was outside the interquartile range of the OR estimated with the gold standard GRS when the percentage of missing SNPs was ≥50% and the OR = exp(*β*) was >1.06.

**Fig 4:**
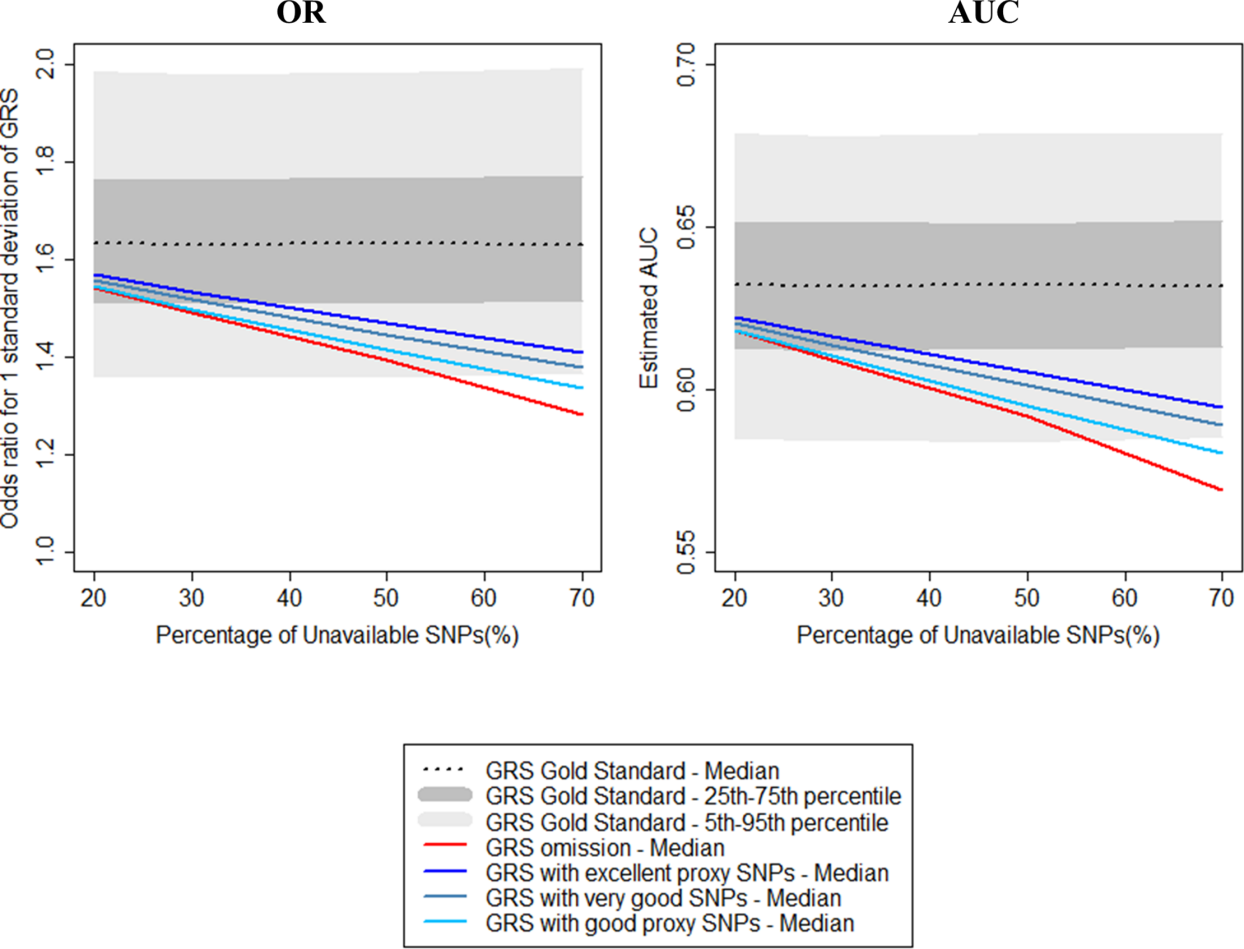
Impact of omission of the unavailable SNPs or replacement by proxy SNPs on effect size estimation and predictive ability of the weighted GRS for the phenotype.

**Fig 5:**
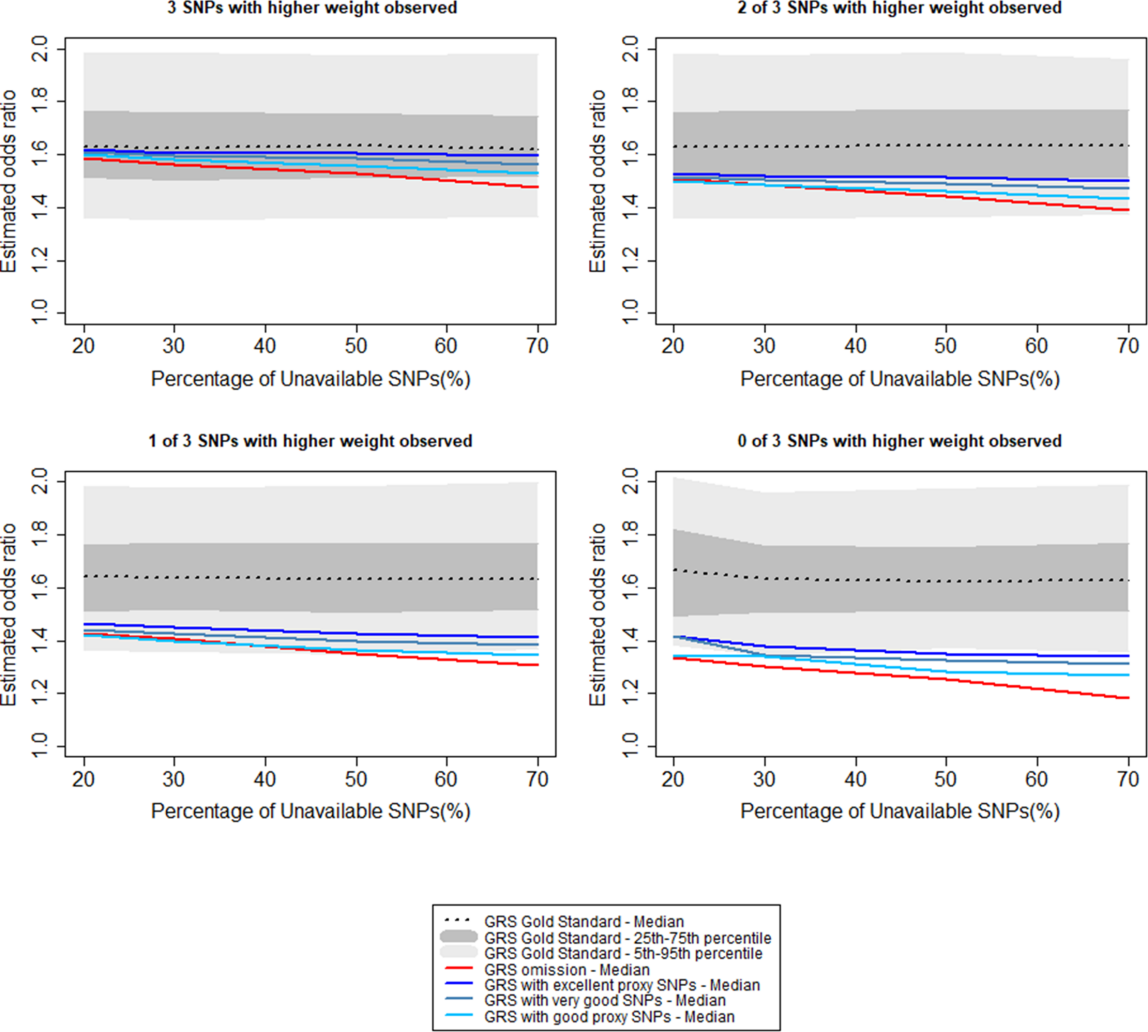
Impact of the unavailability of SNPs corresponding to extreme weights on effect size estimation and predictive ability of the weighted GRS for the phenotype.

Fig 4 shows the estimated OR for a standard deviation increase in the weighted GRS as a function of the percentage of unavailable SNPs when data were simulated using the weighted gold standard GRS. Compared to omission, the proxy approach underestimates the OR to a lesser extent. When 20% of SNPs were unavailable, the median OR estimated for all alternative GRS was within the interquartile range of the gold standard GRS. When 40% of SNPs were unavailable, the median ORs were all outside the interquartile range. Fig 5 illustrates how underestimation is driven by unavailability of the three SNPs with the largest weights. When these three SNPs were included in the GRS, the median ORs for the omission or proxy approaches were within the interquartile range of the ORs estimated with the gold standard GRS when ≤50% of SNPs were unavailable. When only one of the three SNPs was included, independent of approach, median ORs were outside the interquartile range for all proportions of unavailable SNPs studied. No association was observed between the mean MAF of unavailable SNPs and underestimation of the OR. Detailed statistics are available in S3 Table.

**Fig 6:**
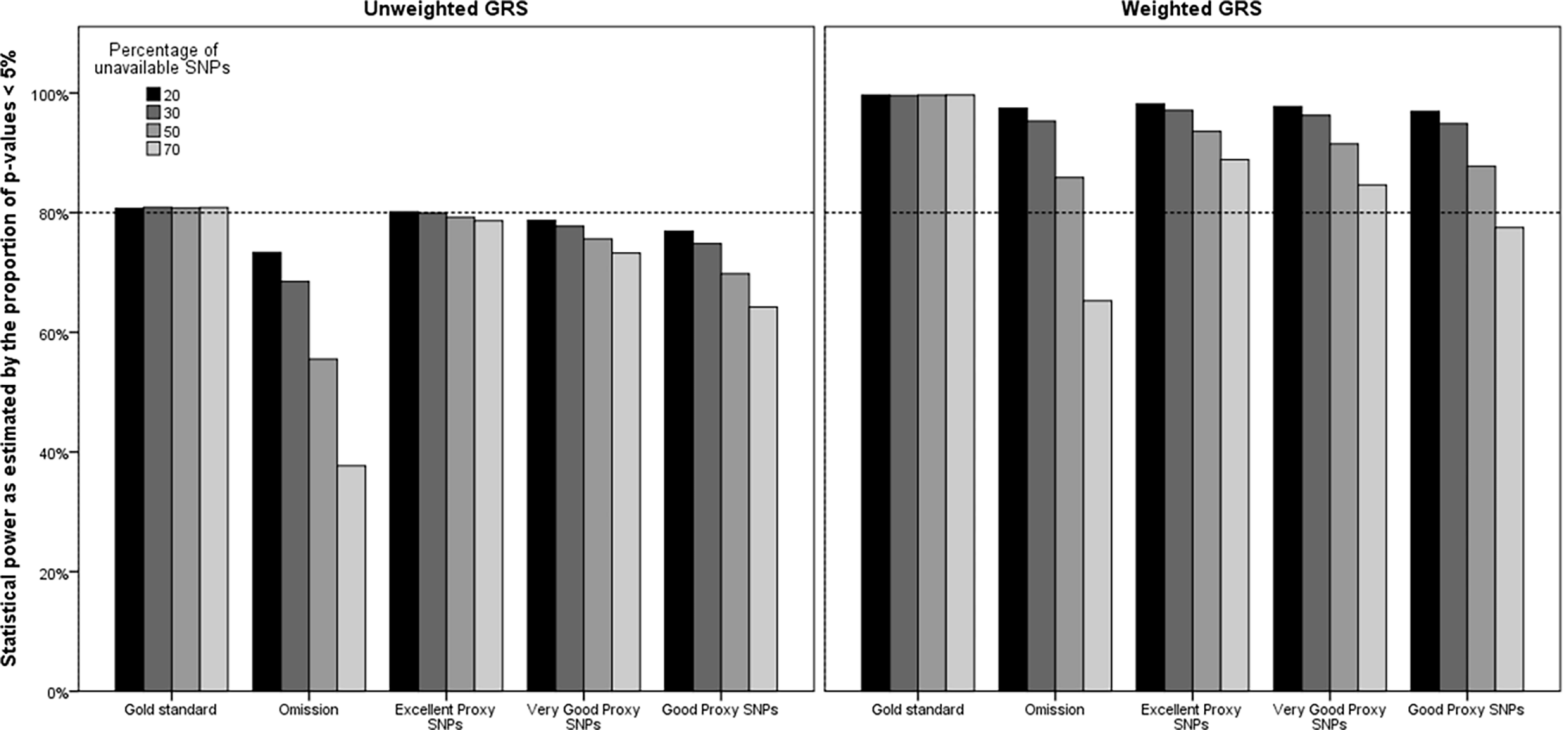
Impact of omission of unavailable SNPs or replacement by proxy SNPs on statistical power to detect the effect size of the weighted GRS for the phenotype.

The statistical power to detect an association between the GRS and the phenotype decreased as the number of omitted SNPs increased (Fig 6). A similar, yet attenuated trend was observed using the proxy SNP approach. While Fig 6 suggests that the statistical power was consistently higher for the weighted GRS than the unweighted GRS, it is a consequence of the values specified in the genetic causal model used to simulate data. Indeed, the strength of the association between the unweighted GRS and the phenotype was weaker than that of the weighted GRS, which adversely affected statistical power. The median OR for the unweighted gold standard GRS ranged from 1.23 to 1.50 depending on the weights used in Eq 2. The median OR of the weighted gold standard GRS, was larger (1.63) and resulted from using the weights reported in Tada et al [6].

### Impact on predictive ability

Investigations of the predictive ability of the weighted and unweighted GRS are reported in Figs 3 and 4. Although the measurement scales differed, the results were identical to those reported for the OR. This is a consequence of the mathematical relation between the log OR and the AUC for a single predictor under the hypothesis of a normal distribution and the homogeneity of variance of groups [27]. In this situation, the AUC is given by:

$$AUC=\phi (\frac{\sigma \times \beta }{\sqrt{2}})$$ (3)

where *ϕ*() is the standard normal cumulative distribution function, *β* is the log OR of coefficient and *σ* is the common variance of groups. In addition, in such cases, the OR of a standardised GRS directly reflects the AUC. Over all scenarios, the difference in the AUC calculated for the median OR with this formula and the median AUC observed over all simulations was less than 0.01.

## Discussion

We undertook a simulation study to investigate the impact of either omitting or using proxy SNPs to construct a GRS when some SNPs are unavailable. Compared to the proxy approach, omission led to larger attenuation of the OR estimating the association between the GRS and the phenotype, smaller predictive ability and more importantly loss of statistical power, even when 70% of SNPs included in the GRS were replaced by proxy SNPs with modest correlation with the unavailable SNPs (0.6< r^2^<0.8). In addition, we found that for GRS that include larger-weight SNPs, predictive ability was driven by whether the SNPs with the largest weights were available. Omitting SNPs with large weights can lead to severe decreases in predictive ability.

Our results are consistent with those of Goldstein et al. [4] who described the performance of large GRS when omitting imputed SNPs based on imputation quality threshold on the performance of large GRS. They reported that omission resulted in attenuation of the relative risk, which became more severe as the number of omitted SNPs increased. Although our findings also suggest that omission leads to attenuation of the OR, they differ from those of Goldstein et al in two ways. First, because Goldstein et al investigated GRS calculated from thousands of SNPs, the impact of omitting a given number of SNPs seemed less severe than in our study for which the number of missing SNPs was a large proportion of the number of SNPs considered. Second, because SNPs were omitted based on the quality of imputation, the effects of omission and quality of imputation could not be disentangled. Our findings suggest that using proxy SNPs led to less severe attenuation of the OR, regardless of the quality of proxy considered.

Unlike our study, Goldstein et al reported that omitting SNPs did not have an impact on the predictive ability of the GRS [4]. However, the AUC reported in Goldstein et al was based on models including both the GRS and clinical risk factors for the phenotype (i.e., coronary heart disease (CHD)). These clinical factors, which corresponded to those used in the Framingham risk score, are known to predict a large proportion of the variation in risk of CHD, with estimates for the AUC ranging from 0.74 in men to 0.77 women [28]. Thus, the genetic contribution to CHD risk in Goldstein et al’s study was relatively small and prevented detection of large changes in the AUC. Because our models did not include any variables other than the GRS, we were able to isolate the effect of omitting SNPs or using proxy SNPs on the performance of the GRS to predict our simulated outcome using the mathematical relationship between the AUC and the OR from univariate logistic models [27].

Regardless of approach used to manage missing SNPs, we observed a slight inverse relationship between the mean MAFs for unavailable SNPs and the correlations between the gold standard GRS and the estimated GRSs. When unavailable SNPs have a small mean MAF, the variance of the number of risk alleles corresponding to the unavailable SNPs is also small. Therefore, the contribution of unavailable SNPs to the gold standard GRS is low, and omitting or replacing them by proxy SNPs has little impact on the GRS.

Strengths of this analysis include the use of real genetic data and a reference panel matched for ethnicity. Because we did not consider other variables than the GRS in our models, our simulations quantified the impact of the omission and proxy approaches on statistical power and predictive ability of the GRS to predict a binary phenotype. Investigating the proxy and omission approaches separately allowed us to distinguish the effect of omitting a SNP from that of replacing it with a proxy SNP.

Limitations of this study include using a GRS with a fixed number of SNPs and only one set of weights in the weighted GRS. Moreover, we used the same weights to generate data from the causal model and to calculate the GRS in each simulated dataset. This is akin to assuming that the weights derived from a discovery sample are the true weights and no error term is included in the data generation. While this is a restrictive assumption, it allowed us to isolate the effect of unavailable SNPs from that of precision in the weights estimation. While we restricted our study to SNPs with MAF ≥ 5%, our results suggest that unavailable SNPs with small MAFs should not have a large impact on the GRS value. Finally, while we did not consider imputation as a method to manage omitted SNPs, we expect that our results pertaining the proxy SNP approach likely apply to imputed SNPs as well because assessing the quality of proxy SNPs is very similar to that of the quality of imputation. Future studies will need to confirm this hypothesis.

## Conclusions

Our study has three practical implications. First, our results suggest that it is generally better to replace unavailable SNPs with proxy SNPs than to omit them from the GRS, particularly when the GRS does not include a very large number of SNPs (as in Goldstein et al.). Second, close attention must be paid to missing SNPs that have relatively high weights in weighted GRS, as failure to use proxy SNPs may significantly impact the performance of the GRS. Third, our results highlight the importance of reporting the number of SNPs that are unavailable when a GRS is computed, as well as the method used to account for the unavailable SNPs. Otherwise, poor performances of GRS that omit several SNPs could be wrongly attributed to failures to replicate the GRS in populations independent from the initial discovery samples.

## Supporting information

S1 Fig Association between the mean of minor allele frequency of missing SNPs and the correlation between the Gold Standard Genetic Risk Score (GRS) and unweighted GRS. (TIF)

S2 Fig Association between the mean of minor allele frequency of missing SNPs and the correlation between the Gold Standard Genetic Risk Score (GRS) and weighted GRS. (TIF)

S1 Table Correlation between the Gold Standard Genetic Risk Score and weighted and unweighted GRS. (DOCX)

S2 Table Correlation between the Gold Standard Genetic Risk Score and weighted and unweighted GRS according to the number of SNPs observed among the SNPs with the biggest weights. (DOCX)

S3 Table Estimated odds ratios for the weighted and unweighted GRS. (DOCX)
