# Supplementary figures and images for: Missing single nucleotide polymorphisms in Genetic Risk Scores: A simulation study
Source: PLoS One. 2018 Jul 19;13(7):e0200630. doi: 10.1371/journal.pone.0200630 (PMC6053141; doi:10.1371/journal.pone.0200630)

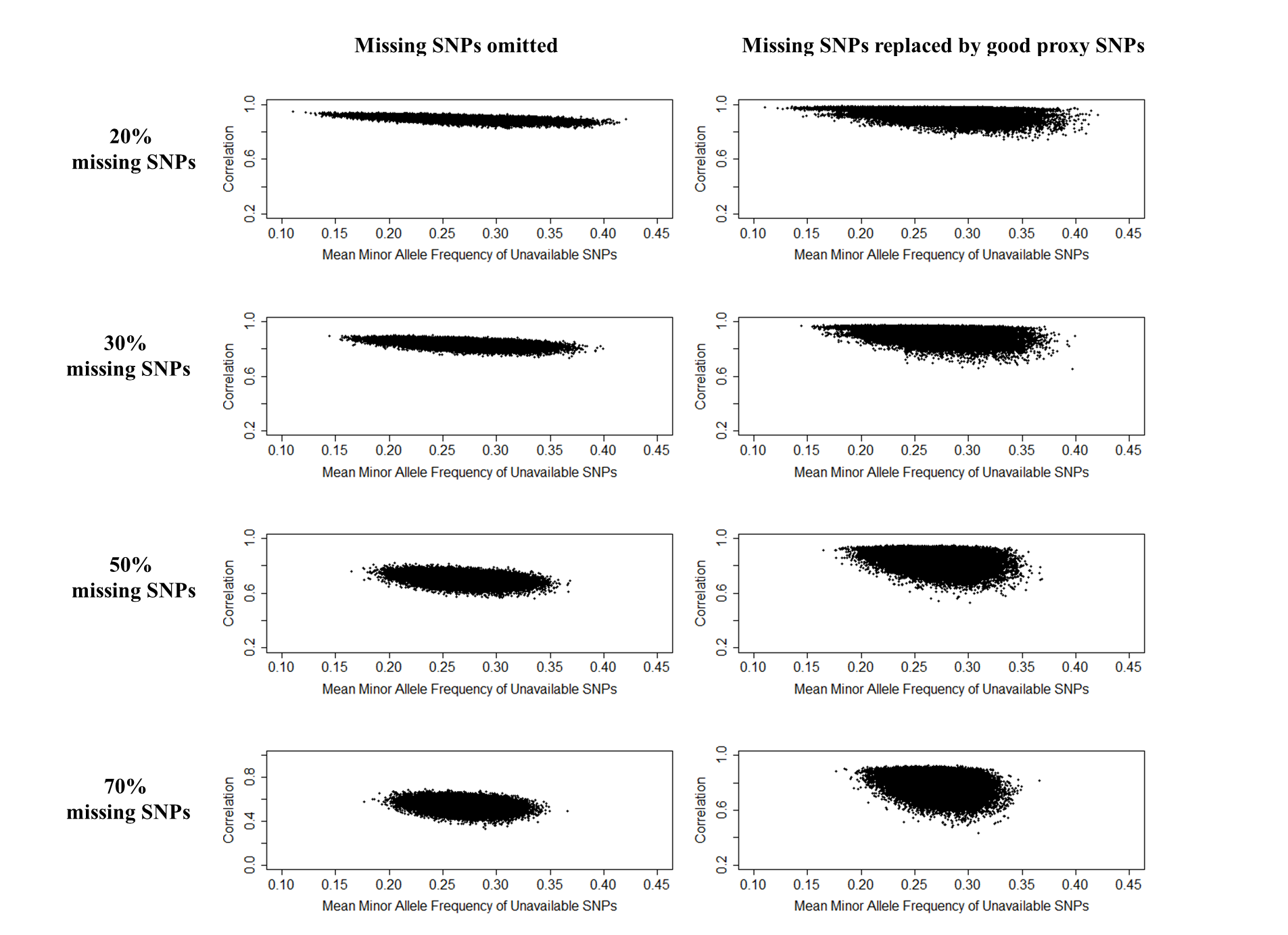

Supplement: S1 Fig — (TIF) [file pone.0200630.s001.tif]

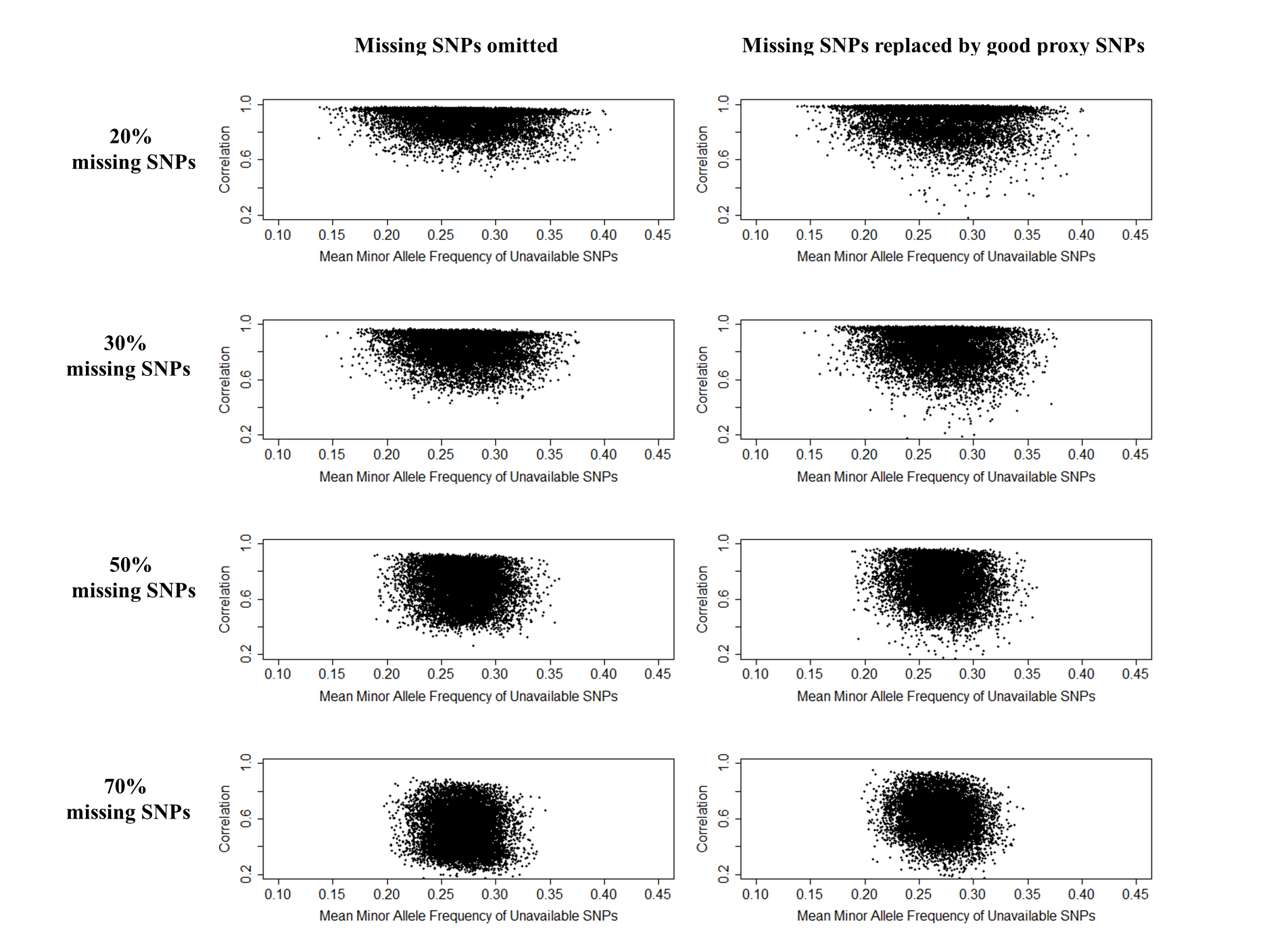

Supplement: S2 Fig — (TIF) [file pone.0200630.s002.tif]
