# Supplementary material for: Missing single nucleotide polymorphisms in Genetic Risk Scores: A simulation study
Source: PLoS One. 2018 Jul 19;13(7):e0200630. doi: 10.1371/journal.pone.0200630 (PMC6053141; doi:10.1371/journal.pone.0200630)
Supplement: S1 Table — (DOCX) [file pone.0200630.s003.docx]

S1Table: Correlation Between the Gold Standard Genetic Risk Score and Weighted and Unweighted GRS

| GRS With … |  | Unavailable SNPs | | | | | | | |
| --- | --- | --- | --- | --- | --- | --- | --- | --- | --- |
|  |  | Unweighted GRS | | | | Weighted GRS | | | |
|  |  | 20% | 30% | 50% | 70% | 20% | 30% | 50% | 70% |
| SNPs available | Median | 0.894 | 0.836 | 0.706 | 0.547 | 0.927 | 0.852 | 0.707 | 0.531 |
|  | 25^th^.75^th^ Percentile | 0.884;0.904 | 0.822;0.850 | 0.685;0.727 | 0.518;0.574 | 0.837;0.954 | 0.765;0.918 | 0.606;0.797 | 0.402;0.645 |
|  | Correlations > 0.8 | 100,0% | 95,2% | 0,0% | 0,0% | 84,3% | 64,6% | 24,4% | 3,1% |
| Excellent proxy SNPs | Median | 0.995 | 0.992 | 0.986 | 0.980 | 0.975 | 0.910 | 0.803 | 0.718 |
|  | 25^th^.75^th^ Percentile | 0.993; 0.996 | 0.989;0.994 | 0.981;0.989 | 0.970;0.984 | 0.864;0.990 | 0.811;0.978 | 0.723;0.900 | 0.630;0.797 |
|  | Correlations > 0.8 | 100,0% | 100,0% | 100,0% | 100,0% | 89,4% | 78,5% | 0,9% | 23,8% |
| Very good proxy SNPs | Median | 0.981 | 0.970 | 0.945 | 0.899 | 0.958 | 0.887 | 0.773 | 0.676 |
|  | 25^th^.75^th^ Percentile | 0.974; 0.985 | 0.930;0.976 | 0.903;0.957 | 0.873;0.936 | 0.847;0.983 | 0.790;0.963 | 0.683;0.868 | 0.583;0.760 |
|  | Correlations > 0.8 | 100,0% | 99,9% | 99,2% | 96,1% | 85,3% | 72,1% | 41,2% | 15,3% |
| Good proxy SNPs | Median | 0.959 | 0.914 | 0.862 | 0.803 | 0.937 | 0.858 | 0.730 | 0.611 |
|  | 25^th^.75^th^ Percentile | 0.920; 0.968 | 0.892;0.948 | 0.821;0.895 | 0.757;0.842 | 0.825;0.972 | 0.759;0.941 | 0.629;0.829 | 0.519;0.707 |
|  | Correlations > 0.8 | 99,8% | 98,5% | 84,5% | 51,8% | 80,4% | 63,7% | 30,7% | 8,4% |
