# Supplementary material for: Missing single nucleotide polymorphisms in Genetic Risk Scores: A simulation study
Source: PLoS One. 2018 Jul 19;13(7):e0200630. doi: 10.1371/journal.pone.0200630 (PMC6053141; doi:10.1371/journal.pone.0200630)
Supplement: S2 Table — (DOCX) [file pone.0200630.s004.docx]

S2 Table: Correlation Between the Gold Standard Genetic Risk Score and Weighted and Unweighted GRS According to the Number of SNPs Observed among the SNPs with the Biggest Weights

| GRS With … | |  | Unavailable SNPs | | | |
| --- | --- | --- | --- | --- | --- | --- |
|  |  | Number of SNPs | 20% | 30% | 50% | 70% |
| SNPs available | Median | 0/3 | 0,60 | 0,57 | 0,48 | 0,37 |
|  | 25^th^.75^th^ Percentile |  | 0,57;0,64 | 0,54;0,6 | 0,44;0,52 | 0,33;0,41 |
|  | Median | 1/3 | 0,74 | 0,71 | 0,64 | 0,57 |
|  | 25^th^.75^th^ Percentile |  | 0,69;0,78 | 0,65;0,76 | 0,58;0,69 | 0,50;0,63 |
|  | Median | 2/3 | 0,86 | 0,83 | 0,77 | 0,70 |
|  | 25^th^.75^th^ Percentile |  | 0,81;0,9 | 0,78;0,87 | 0,72;0,81 | 0,65;0,75 |
|  | Median | 3/3 | 0,95 | 0,93 | 0,88 | 0,82 |
|  | 25^th^.75^th^ Percentile |  | 0,94;0,96 | 0,92;0,94 | 0,86;0,89 | 0,80;0,85 |
| Excellent proxy SNPs | Median | 0/3 | 0,73 | 0,68 | 0,63 | 0,61 |
|  | 25^th^.75^th^ Percentile |  | 0,67;0,81 | 0,62;0,75 | 0,59;0,69 | 0,57;0,66 |
|  | Median | 1/3 | 0,79 | 0,78 | 0,75 | 0,73 |
|  | 25^th^.75^th^ Percentile |  | 0,73;0,85 | 0,71;0,83 | 0,69;0,80 | 0,67;0,78 |
|  | Median | 2/3 | 0,88 | 0,87 | 0,85 | 0,83 |
|  | 25^th^.75^th^ Percentile |  | 0,83;0,94 | 0,82;0,93 | 0,80;0,91 | 0,79;0,89 |
|  | Median | 3/3 | 0,99 | 0,98 | 0,97 | 0,96 |
|  | 25^th^.75^th^ Percentile |  | 0,98;0,99 | 0,98;0,99 | 0,96;0,98 | 0,95;0,97 |
| Very good proxy SNPs | Median | 0/3 | 0,70 | 0,65 | 0,60 | 0,57 |
|  | 25^th^.75^th^ Percentile |  | 0,63;0,79 | 0,58;0,71 | 0,55;0,65 | 0,52;0,62 |
|  | Median | 1/3 | 0,77 | 0,75 | 0,72 | 0,69 |
|  | 25^th^.75^th^ Percentile |  | 0,70;0,83 | 0,68;0,8 | 0,65;0,77 | 0,63;0,75 |
|  | Median | 2/3 | 0,86 | 0,85 | 0,83 | 0,80 |
|  | 25^th^.75^th^ Percentile |  | 0,81;0,93 | 0,8;0,91 | 0,77;0,89 | 0,75;0,86 |
|  | Median | 3/3 | 0,98 | 0,97 | 0,95 | 0,93 |
|  | 25^th^.75^th^ Percentile |  | 0,97;0,99 | 0,96;0,98 | 0,93;0,96 | 0,91;0,94 |
| Good proxy SNPs | Median | 0/3 | 0,64 | 0,59 | 0,54 | 0,51 |
|  | 25^th^.75^th^ Percentile |  | 0,57;0,7 | 0,53;0,66 | 0,49;0,6 | 0,45;0,56 |
|  | Median | 1/3 | 0,74 | 0,71 | 0,67 | 0,63 |
|  | 25^th^.75^th^ Percentile |  | 0,67;0,8 | 0,64;0,77 | 0,6;0,73 | 0,56;0,69 |
|  | Median | 2/3 | 0,84 | 0,82 | 0,79 | 0,75 |
|  | 25^th^.75^th^ Percentile |  | 0,79;0,91 | 0,77;0,89 | 0,73;0,85 | 0,69;0,81 |
|  | Median | 3/3 | 0,97 | 0,95 | 0,92 | 0,89 |
|  | 25^th^.75^th^ Percentile |  | 0,96;0,98 | 0,94;0,97 | 0,9;0,94 | 0,86;0,91 |
