# Supplementary material for: Missing single nucleotide polymorphisms in Genetic Risk Scores: A simulation study
Source: PLoS One. 2018 Jul 19;13(7):e0200630. doi: 10.1371/journal.pone.0200630 (PMC6053141; doi:10.1371/journal.pone.0200630)
Supplement: S3 Table — (DOCX) [file pone.0200630.s005.docx]

S3 Table: Estimated Odds Ratios for the Weighted and Unweighted GRS

| GRS With … |  | Unavailable SNPs | | | | | | | |
| --- | --- | --- | --- | --- | --- | --- | --- | --- | --- |
|  |  | Unweighted GRS | | | | Weighted GRS | | | |
|  |  | 20% | 30% | 50% | 70% | 20% | 30% | 50% | 70% |
| SNPs available | Median | 1,36 | 1,36 | 1,36 | 1,36 | 1,63 | 1,63 | 1,63 | 1,63 |
|  | 25^th^.75^th^ Percentile | 1,25;1,48 | 1,25;1,48 | 1,25;1,48 | 1,25;1,48 | 1,51;1,76 | 1,51;1,76 | 1,51;1,76 | 1,51;1,77 |
| Excellent  proxy SNPs | Median | 1,31 | 1,29 | 1,24 | 1,18 | 1,54 | 1,49 | 1,39 | 1,28 |
|  | 25^th^.75^th^ Percentile | 1,21;1,43 | 1,19;1,40 | 1,15;1,34 | 1,10;1,27 | 1,43;1,67 | 1,38;1,62 | 1,28;1,52 | 1,18;1,40 |
| Very good proxy SNPs | Median | 1,36 | 1,35 | 1,35 | 1,34 | 1,57 | 1,53 | 1,47 | 1,41 |
|  | 25^th^.75^th^ Percentile | 1,25;1,48 | 1,24;1,48 | 1,24;1,47 | 1,24;1,47 | 1,45;1,70 | 1,41;1,67 | 1,35;1,60 | 1,30;1,53 |
| Good proxy SNPs | Median | 1,35 | 1,34 | 1,33 | 1,31 | 1,56 | 1,52 | 1,44 | 1,38 |
|  | 25^th^.75^th^ Percentile | 1,24;1,47 | 1,23;1,46 | 1,22;1,44 | 1,21;1,43 | 1,44;1,69 | 1,4;1,65 | 1,33;1,57 | 1,27;1,50 |
